# Cost volume profit analysis for full paying patient services in Malaysia: A study protocol

**DOI:** 10.1371/journal.pone.0294623

**Published:** 2023-11-21

**Authors:** Malindawati Mohd Fadzil, Sharifa Ezat Wan Puteh, Azimatun Noor Aizuddin, Zafar Ahmed, Nor Asiah Muhamad, Abdul Aziz Harith

**Affiliations:** 1 Department of Community Medicine, University Kebangsaan Malaysia Medical Centre, Cheras, Kuala Lumpur, Malaysia; 2 Department of Community Medicine and Public Health, Faculty of Medicine, University Malaysia Sarawak, Sarawak, Malaysia; 3 Department of Social Work, Education and Community Wellbeing, Faculty of Health and Life Sciences, Northumbria University, Newcastle-upon-Tyne, United Kingdom; 4 Evidence Based Healthcare Medicine Sector, National Institutes of Health, Ministry of Health, Shah Alam, Selangor, Malaysia; 5 Occupational Health Research Centre, Institute for Public Health, Ministry of Health Malaysia, Shah Alam, Selangor, Malaysia; Universiti Malaysia Sabah, MALAYSIA

## Abstract

Dual practice within public hospitals, characterised by the concurrent provision of public and private healthcare services within public hospitals, has become a widespread phenomenon. With the participation of selected public hospitals, dual practice within public hospitals, also known as Full Paying Patient services, was an initiative the Ministry of Health Malaysia took in 2007 to retain senior specialist physicians in Malaysia. The revenue generated from the Full Paying Patient services aims to provide an avenue for public sector specialists to supplement their incomes while alleviating the Government’s burden of subsidising healthcare for financially capable individuals. However, the effectiveness of Full Paying Patient services in recouping service delivery costs and yielding a profit is still uncertain after 16 years of implementation. This study is designed to evaluate the impact of Full Paying Patient inpatient services volume, revenue, and cost on profit versus loss at selected hospitals from 2017 to 2020. From the perspective of healthcare providers, we plan to perform a cost volume profit analysis. This analysis enables us to determine the break-even point, at which total revenues match total costs, along with no-loss and no-profit thresholds for Full Paying Patient services. This study has the potential to provide insights into how variations in service volume, cost, and pricing impact healthcare providers’ profitability. It also offers critical financial information regarding the volume of services required to reach the break-even point. A comprehensive understanding of service volume, cost and pricing is imperative for making informed decisions to fulfil the objectives and ensure the sustainability of the FPP services.

## Introduction

The expansion of private healthcare globally has led to a significant loss of specialist physicians from the public healthcare sector, causing an imbalanced workforce distribution. The diminishing public workforce is concerning, given that senior specialist physicians play a vital role in complex procedures and optimal treatment outcomes [1]. By sharing their expertise with junior physicians, they ensure the sustainable delivery of high-quality, accessible, and equitable healthcare [2]. Without these senior specialist physicians, the ability of public hospitals to provide quality care and expertise is significantly weakened. The loss of specialist physicians from the public sector will have far-reaching negative consequences for the entire healthcare system. Therefore, authorising specialist physicians in the public healthcare sector to engage in dual practice is a common policy response by both low and high-income countries to address attrition [3]. Dual practice within a public hospital setting (DPH) is one form of dual practice, with physicians treating private patients alongside public patients in the same public hospital setting but outside the public service’s operating hours to provide quick access for an additional fee [4].

DPH has been practised in various countries, including Austria, Ireland, Italy, Australia, the United Kingdom (UK), France, Germany [4, 5], Indonesia [6], Vietnam [7], Ethiopia [8], and Uganda [9]. Existing literature highlights both positive and negative outcomes of DPH. Adverse effects include specialist physicians devoting more attention to private patients, longer waiting times, increased rates of absenteeism and neglecting duties, prioritising profit-making cases, and decreased trust in the public healthcare sector [5, 10, 11]. Conversely, DPH had beneficial impacts such as enhancing the appeal of the public healthcare sector to potential employees [5, 11, 12], improving the retention of healthcare professionals, improving access to quality healthcare services [8, 13], and providing patients with treatment options [5, 14].

DPH also provides an alternative financing approach for the healthcare system. It generates additional income within public hospitals [5, 11, 12, 14, 15]. The revenue could be used to expand public healthcare services [16], upgrade public healthcare facilities [17], supplement hospital operating expenses, offer staff incentives, and invest in health promotion, preventive care [18], training, research, equipment, and staff [11].

Nevertheless, the effects of dual practice on healthcare expenses and efficiency remain unclear [10]. Insufficient financial planning, inadequate cost and revenue monitoring, and inappropriate pricing adversely influence healthcare delivery costs. The Victorian Auditor-General’s report on DPH practice in Australia revealed a lack of evidence that fees paid by specialists to facilities accurately reflect the actual value of public resources used and may not cover actual costs [11]. A report from the UK National Health Service (NHS) indicated significant income generation from private patients within public hospital settings, growing from £511 million to £596 million (USD 652 million to USD 760 million) over four years [12]. While proper tracking of revenues generated from DPH was lacking, four NHS hospitals recorded substantial losses of up to £18 million (USD 23 million) over six years. Thus, it is still unclear whether DPH revenue offsets service delivery costs. Moreover, concerns persist regarding the unequal allocation of important resources from public hospitals to private practice leading to unintended losses for the already resource-constrained public healthcare sector [12].

### Background of dual practice within public hospitals in Malaysia

Malaysia, an upper-middle-income country, operates a dual-tier national healthcare system encompassing a government-led public healthcare sector and a concurrently existing private healthcare system. The public healthcare system relies mainly on general taxation, co-existing alongside the market-oriented private sector. From 1997 to 2019, Malaysia’s total health expenditure (TEH) fluctuated between 3.04% and 4.30% of the Gross Domestic Product (GDP) [19]. Public health expenditures stood at 2.58% in 2020, whereas other middle- and high-income countries allocated at least 4% [20]. In 2019, the public health expenditure constituted 52% of the TEH [19]. Notably, as high as 99% of health expenditure in the public sector is under government subsidy, while patients contributed only 1% to 2% of the total cost as co-payment [21].

Malaysian residents are entitled to a wide range of highly subsidised healthcare services, including inpatient and outpatient care within the public healthcare system, with nominal user charges [22]. Patients seeking care from specialist outpatient clinics are only charged MYR 5.00 (equal to USD 1.08), encompassing all consultations, investigations, and medications [23]. This framework, however, excludes noncitizens and Full Paying Patient services [24].

In response to economic challenges, resource constraints and an alarming attrition rate of specialists to the private sector, the Ministry of Health Malaysia (MOH) introduced the Full Paying Patient (FPP) services in 2017. This initiative serves as a retention program for senior specialist physicians to earn supplementary income and generate revenue to alleviate the Government’s financial burden of subsidising healthcare for financially capable patients [25]. FPP services have proven effective in enhancing the retention of senior specialist physicians within MOH hospitals and reducing the Government’s financial burden [26]. FPP patients are charged the full price without government subsidies. The Fees (Medical) (Full Paying Patient Service) Order 2007 (FPP Fee Order) specifies the types and rates of FPP fees [25]. They are allowed to select their preferred specialists and granted priority access to treatment and first-class wards or equivalent, based on the hospital’s resources and facilities [27].

Revenue generated from FPP is apportioned between FPP specialists and the Government at a regulated percentage based on the type of service. For example, FPP specialists receive 100% of revenue from procedures, while the Government gains the 100% from consumables [25]. Notably, FPP services have generated over MYR 74.3 million (USD 16.4 million) between 2012 and 2017, with a portion of this income being redirected to cover subsidies in public healthcare [26].

A study of two hospitals with FPP services revealed an increasing revenue trend from 2007 to 2016. However, these values were 16% to 52% lower than the estimated costs between 2014 and 2016 [28]. The cost calculation was performed using the total revenue and estimated costs associated with the top ten frequently encountered International Statistical Classification of Diseases and Related Health Problems–10^th^ Revision (ICD-10) diagnosis codes across the three most frequently visited clinical departments of each hospital, namely Obstetrics and Gynaecology, Ophthalmology, and Orthopaedics [28]. The study excluded other clinical departments providing FPP services and assumed that all cases had Class 1 severity of illness (without co-morbidities and/ or complications). In general, complicated cases involving co-morbidities, complications, procedures, or surgeries yield higher revenue than uncomplicated cases [29]. The results of this study may not accurately reflect the profitability of FPP service, and it may be necessary to conduct further research in this area. Additionally, the National Audit Department of Malaysia reported that charges for FPP services had not been updated since 2007, with many newly introduced fees not being incorporated [26]. Therefore, this situation raises concerns that charges may fall short of covering service costs. As a result, leaving the potential for cost recovery or profit generation through FPP services in question. To validate the practicality of FPP services in lowering healthcare costs in Malaysia and to ensure its sustenance, it is imperative the generation a surplus of revenue over service costs.

In the accounting domain, profit is defined as the remainder following the settlement of all costs [30]. In the healthcare sector, numerous not-for-profit organisations use the phrase ’surplus of revenues’ over the word ‘profit’ to describe their financial performance [31]. Whether an entity operates as a for-profit or not-for-profit organisation, financial considerations are vital in the decision-making process [32]. There is a risk of profit deficits or losses from the FPP service that will necessitate a cross-subsidy from the healthcare budget intended for patients in the public healthcare sector, indicating that the FPP service is not sustainable and negatively affecting public service equity. According to Suwandono et al. [33], public hospitals investing in commercial beds require effective management and accounting systems to monitor and control expenditures and set fees to recoup the expenses. However, insufficient revenue generation has increasingly been identified as a key problem hindering hospital-wide quality improvement initiatives, such as FFP services [34]. Cost volume profit (CVP) analysis is an outstanding method for analysing FPP service financial performance and costs since it supports the analysis of cost recovery and profitability [35].

### The concept of cost volume profit (CVP) analysis

CVP analysis is also known as profit or break-even analysis in financial evaluation. Originally, Hess [36] and Mann [37] presented the CVP model from a managerial accounting perspective [38, 39]. In this method, changes in costs, volume of service, and price are analysed and estimated for profit [30]. CVP analysis divides total cost into fixed and variable costs to be examined [40]. Fixed costs are not related to the volume of services delivered within a specific period of time [41]. For example, patient volume does not affect staff salaries and maintenance costs [42]. While variable costs vary with the output volume of a service, for instance, supplies and medications costs fluctuate with patient volume [30].

The contribution margin (CM) is key to calculating CVP. CM is the difference between revenue per unit and variable costs per unit for each unit of volume, which covers fixed costs and ultimately flows to profit [30]. Break-even analysis (BEA) is an essential component of CVP, which calculates the volume of services needed to cover costs and identifies the break-even point (BEP). BEP is the point at which total revenues equal total costs resulting in zero operating income, or the point at which the organisation must cover all fixed expenses since no profit is generated [41].

The literature mainly discusses CVP analysis for manufacturing and business industries [43–46]. Meanwhile, several healthcare-related CVP analysis examples include the determinants of unit analysis and hospital cost structure [40], profitability analysis of mechanical ventilators [47], cost advantage comparison of two parenteral nutrition approaches [48], the CVP structure of teaching and non-teaching hospitals [49], and the estimated national impact of pricing policy changes on pharmacies gross profit [50]. It has been emphasised that CVP analysis is particularly essential for non-profit organisations. CVP analysis should be conducted in non-profit organisations since financial information is valuable in planning and making decisions for effective resource management [51]. This method can also guide service and non-profit organisations to maintain service outputs even with reduced budgets [41].

### Assumptions of CVP analysis

Similar to most financial models, CVP analysis relies on simplifying assumptions to reduce the complexity of input and output variables to facilitate decision-making. To fully comprehend the function of a financial model and its usefulness, it is crucial to understand its assumptions and their role in the decision-making process. As described by Datar and Rajan [41], there are several underlying assumptions underlying the basic CVP model:

1. The total cost must be able to be divided into fixed and variable components.
2. The behaviour of total costs and total revenues are linear in nature in relation to the volume of services delivered within a relevant range of time periods.
3. Variable cost per unit, revenue per unit, and total fixed costs can be identified and will remain constant within a relevant range of time periods.
4. The changes in total costs and total revenues are only affected by changes in the quantities of services delivered. The volume will be the only revenue driver and cost driver.

The basic format of CVP analysis is meant for a single service or product. However, most organisations have more than one diverse service (or product), such as hospitals offering various services. CVP analysis assumes that if a range of services is delivered or products are sold, sales will follow a predetermined sales mix [52]. Sales mix or product mix refers to the number (or proportion) of products (or services) that make up an organisation’s total sales or services [41]. In the CVP analysis of multi-services, the assumption is that services are delivered according to a constant sales mix irrespective of volume [41, 52]. Based on this assumption, the weighted average contribution margin can be calculated [31]. In simple terms, the weighted average contribution margin reflects the contribution margin for each product compared to the share of sales attributable to each product.

### Significance of study

Several studies have demonstrated that FPP patients and revenue have increased since the implementation of the service more than a decade ago [26, 53]. Driven by this encouraging result and the commitment to retain more specialists in the public sector, the MOH has developed plans to expand the FPP service throughout the country. This has been one of the top priorities in the MOH Medical Programme’s Strategic Framework for the 12^th^ Malaysian Plan (2021–2025) [54]. To ensure the success of FPP service expansion and enhance existing service delivery, it is understood that the MOH is currently revising the FPP policies and guidelines, including the outdated FPP charges [26, 54]. Though there has been a high level of preparation and planning, the financial evaluation of the service and the potential surplus of revenue generated from it remain unexplored. According to the experiences of other countries, this type of dual practice may negatively affect public healthcare resources due to poor financial planning, inadequate monitoring of cost and revenue, and unreasonable pricing. It may result in valuable resources being diverted from the public healthcare system to private practices, resulting in unintended losses [12] and public mistrust of the national healthcare system [5].

In the public healthcare sector, revenues, prices, volume of services, and costs must be managed strategically due to their interdependence, which determines profit and loss. This study aims to evaluate the impact of FPP inpatient service volumes, revenues, and costs on the profit versus loss of selected MOH FPP hospitals from 2017 to 2020. The relationship between these factors in generating operating income is vital for healthcare programs’ financial health, efficiency, and sustainability. Valuable financial information from the study will guide policymakers, healthcare managers, hospital managers and key stakeholders in strategic decision-making for the expansion and maintenance of the service. This financial evaluation will illuminate the impact of current outdated procedures and treatment charges on revenue collection and profit generation. This analysis will be essential to guide the policymaker in revising the FPP service charges. From a practical perspective, the CVP method can add beneficial skills for healthcare managers and policymakers in making daily decisions and even planning for future levels of service volume and revenue required to avoid losses to the public healthcare sector.

### Objectives

This study aims to evaluate the impact of FPP inpatient services volume, revenue, and costs on profit versus loss at four selected FPP hospitals between 2017 and 2020. This study will determine the level of service needed to break-even and whether the FPP service’s service charges are sufficient to generate revenues to cover service delivery costs while generating a surplus of revenue (profit). In addition, this study aims to illustrate an overview of the trend in FPP services volumes, revenues, and costs for four selected MOH hospitals from 2017 to 2020.

### Research questions

1. What is the trend in FPP inpatient services volume, revenue, and cost at four MOH hospitals between 2017 and 2020?
2. What is the service volume required for FPP inpatient services to break-even at four selected MOH hospitals from 2017 to 2020?
3. Is the volume of FPP inpatient services delivered by the four selected MOH hospitals sufficient to produce profits between 2017 and 2020?
4. How much profit (or loss) was generated from FPP inpatient services at four selected MOH hospitals from 2017 to 2020?

## Methods

### Study design and area

This quantitative, cross-sectional financial analysis from a healthcare provider’s perspective uses the CVP analysis approach. Secondary data analysis will be conducted to evaluate the impact of three variables: volume, revenue, and cost on the FPP inpatient service’s profit in four selected MOH FPP hospitals. This study will include all inpatient cases from all clinical departments involved in FPP inpatient services. The MOH Malaysian Diagnosis-Related Groups (MalaysianDRG) Casemix system will be used to determine individual case DRG based on actual clinical data and severity of illness (SOI) levels. We will use the casemix costs (MalaysianDRG CGW 2017, 2018, 2019, 2020) and the actual patient charges.

### Study setting

We will conduct this study in public hospitals implementing Full Paying Patient (FPP) services under the Ministry of Health Malaysia (MOH). Before commencing the study, a preliminary investigation revealed that as of December 2020, ten public hospitals in Malaysia offered FPP services [26]. It was also discovered that the MOH implemented the MalaysianDRG Casemix system in selected public hospitals in stages from 2010 onwards, intending to provide health managers and policymakers with healthcare cost information [55]. This study will utilise inpatient data from the MOH MalaysianDRG Casemix system, which captures actual clinical information and the delivery cost of all FPP inpatient cases from 2017 to 2020. This system is fundamental to our research as comprehensive cost data is unavailable [23]. Therefore, the selected FPP inpatient cases must have been discharged from hospitals equipped with this system since 2017. Table 1 shows each FPP hospital’s MalaysianDRG Casemix system availability.

**Table 1:** MalaysianDRG Casemix system availability at ten MOH FPP hospitals.

Hospital	FPP implementation	MalaysianDRG Casemix system availability	Complete Casemix data from 2017 to 2020
Putrajaya	August 2007	Available since 2020	No
Selayang	August 2007	Not available	No
Sarawak Heart Centre	January 2015	Available since 2014	Yes
Queen Elizabeth II	February 2015	Not available	No
Sultan Ismail	June 2015	Available since 2018	No
Ampang	June 2015	Not available	No
Sungai Buloh	September 2015	Not available	No
Pulau Pinang	November 2015	Available since 2013	Yes
Serdang	June 2016	Available since 2010	Yes
Sultanah Aminah	November 2016	Available since 2014	Yes

Source: Casemix Subunit, Medical Services and Management Unit, Medical Development Division [56]

We have selected four MOH FPP public hospitals as the study setting: Serdang Hospital, Pulau Pinang Hospital, Sultanah Aminah Hospital, and Sarawak Heart Centre. These four hospitals are from different regions of Malaysia: the central, northern, southern, and Sarawak zones. Except for Sarawak Heart Centre, multidisciplinary clinical services are provided by the selected FPP hospitals for public patients. Sarawak Heart Centre is a special medical institution that provides specifically identified specialties. All these four hospitals are also part of the regional Cardiothoracic Centres under the MOH. Table 2 shows the profiles of four selected MOH FPP hospitals and the main subspecialties involved in FPP services. Note that the provision of FPP services is not universal across all specialties in these hospitals but instead implemented in phases depending on their capacity and capability.

**Table 2:** Profiles of the four selected MOH FPP hospitals.

Hospital	Type of hospital	Total no. of beds	Clinical departments involved in FPP inpatient service
Sarawak Heart Centre	Special medical institution	128	Cardiology, Cardiac Anaesthesiology and Perfusion, Cardiothoracic Surgery
Pulau Pinang	State hospital	1,158	Cardiology, Dermatology, Diagnostic Radiology, General Surgery, Neurology, Obstetrics and Gynaecology, Ophthalmology, Otorhinolaryngology, Orthopaedic, Pathology, Paediatric, Anaesthesiology and Intensive Care, Cardiothoracic Surgery, Urology, Cardiac Anaesthesiology and Perfusion
Serdang	Major specialist hospital	763	Cardiology, Ophthalmology, Anaesthesiology and Intensive Care, Cardiothoracic Surgery, Cardiac Anaesthesiology and Perfusion, Pathology, Radiology
Sultanah Aminah	State hospital	1,206	Obstetrics and Gynaecology, General Medicine, Plastic Surgery, Nuclear Medicine, Pathology, Radiology, Anaesthesiology, and Intensive Care

Source: Medical Development Division, Ministry of Health Malaysia [57]

### Study population

The study population included all FPP inpatient cases at ten MOH FPP public hospitals in Malaysia from 2017 to 2020. The FPP Service Subunit in the Medical Development Division of the MOH reported a total of 23,997 inpatient cases between 2017 and 2020.

### Inclusion criteria

The inclusion criteria for this study are as follows:

1. FPP inpatient cases discharged from four selected hospitals between 2017 and 2020.
2. FPP inpatient cases in the MOH MalaysianDRG Casemix system in four selected FPP hospitals between 2017 and 2020.
3. FPP inpatient cases with recorded hospital charges in the Revenue Unit database at four selected FPP hospitals between 2017 and 2020.

### Exclusion criteria

The exclusion criteria for this study are as follows:

1. FPP cases attending daycare, outpatient department, or emergency department.
2. FPP inpatient cases with incomplete casemix data in the MOH MalaysianDRG Casemix system.
3. FPP inpatient cases with incomplete charge records in the hospitals’ Revenue Unit database.

### Sample size calculation and sampling technique

The minimum sample size required for this study is estimated using the Krejcie and Morgan sampling method formula with a 95% confidence level. Krejcie and Morgan determined the sample size using the following formula [58]:

$$s=\frac{X^{2}NP(1-P)}{d^{2}(N-1)+X^{2}P(1-P)}$$

$$s=\frac{3.841\times 23,997\times 0.50(1-0.50)}{0.05^{2}(23,997-1)+[3.841\times 0.50(1-0.50)]}$$

s=379

Where:

s = required sample size

X^2^ = table value of chi-square for one degree of freedom at the desired confidence level (3.841)

N = population size (23,997)

P = population proportion (assumed to be .50 since this would provide the maximum sample size)

d = degree of accuracy expressed as a proportion (.05)

According to Krejcie and Morgan’s table for determining sample size, for a given population of 23,997, a minimum sample size of 379 would be required to represent a cross-section of the population. Samples will be selected by multistage cluster sampling.

### Study tools

Standardised comprehensive template forms will be utilised for this study using Microsoft® Excel® for Microsoft 365 MSO, Version 2205 (Microsoft Corporation, USA). In addition, there will be a guide for completing the form. We prepared the templates based on literature review and expert opinions. In developing these templates, we conducted multiple visits to FPP Service Unit, Casemix Unit, and Revenue Unit in study hospitals. This was to gather information on the data needed for the cost analysis. Expert opinions were also sought from the FPP Service Subunit and Casemix Subunit at MOH headquarters. The complete templates consist of five sections:

1. Patient demographic and encounter data: age, gender, discharge date, primary specialty, primary subspecialty, and length of stay.
2. Patient casemix clinical data: main diagnosis, International Statistical Classification of Diseases and Related Health Problems–10^th^ Revision (ICD-10) code, main procedure, International Classification of Diseases–9^th^ Revision–Clinical Modification (ICD-9-CM) code, Casemix Major Diagnostic Categories (MDC) code, Diagnosis Related Group (DRG) code, type of case (surgical or medical), and the severity of illness (SOI 1, 2, or 3: without, with-, or with major co-morbidities and/or complications).
3. Patient charge data: the total fee charged to the patient upon discharge for all services provided during the stay.
4. Patient casemix costing data: national cost group weight (CGW), hospital CGW, national base rate, hospital base rate, casemix cost per case, and casemix cost per patient day for each DRG.
5. Hospital expenditure data: annual total hospital cost data categorised under specific expenditure codes according to PS 1.1 Classification of Revenue and Expenditure Codes, a circular of the Treasury of Malaysia [59]. For example, code 10,000 for staff emoluments.

All this information is not currently accessible from a centralised data centre within the MOH. The data will be collected from three different departments at four participating hospitals: FPP Service Unit, Casemix Unit, and Revenue Unit, and from two other sources at the national level: MOH FPP Service Subunit and MOH Casemix Subunit.

### The CVP analysis

The study outcome will include FPP inpatient service profit and volume required to break-even. To perform a CVP analysis for this study, seven steps will be involved: 1) determine the service volume, 2) determine the total revenue of the service, 3) determine the service’s operating costs, 4) determine the fixed and variable costs, 5) calculate the profit of the service, 6) determine the contribution margin of the service based on revenue per unit and variable costs per unit, and 7) identify the service volume needed to break-even for FPP inpatient services. These steps will be repeated for all the data from 2017 to 2020 for the selected FPP hospitals. In this study, FPP service profit is the surplus of revenues over total costs, whereas a negative profit indicates a loss [30].

#### The volume of FPP inpatient service

Volume refers to the number of services delivered or completed. The number of patient days is used in this study to measure the volume of services delivered. The number of patient days refers to the total number of days for all patients who were admitted for an episode of care [60]. It is the basic measure of how many services a hospital or clinical department provides. Initially, the hospital information systems of selected FPP hospitals will be used to identify FPP inpatients discharged from 2017 to 2020, and their details will be collected (date of discharge, age, gender, primary specialty, and subspecialty in charge of the patient, and length of hospital stay). Following this, each hospital’s total annual FPP inpatient days will be calculated to determine the volume of FPP inpatient services it provides yearly. Each clinical department’s volume of FPP inpatient services will be calculated by adding the total number of FPP inpatient days in each department.

#### Revenue of FPP inpatient service

Revenue refers to the income received by the hospital. Revenue per patient is the actual charges billed to FPP patients for the admission episode. It will be retrieved from the Revenue Unit in the selected hospitals. The charges are based on the FPP Fee Order 2007, which includes registration fees, consultation fees, hospitality (room) charges, procedure or treatment fees, laboratory tests, medical imaging, consumables, and medications given to the patient during the hospital stay [25, 27]. The total revenue for FPP inpatient services will be reflected in the sum of all individual revenue per patient. The average revenue per patient day will also be calculated for each clinical department involved.

#### Operating costs of FPP inpatient service

The Casemix system has two main components: disease classification and costing. The MalaysianDRG Casemix system uses the top-down costing method [61]. The MalaysianDRG Casemix system assigns diagnostic codes based on the ICD-10, whereas procedural codes are assigned based on the ICD-9-CM to generate DRG codes for all patients [61, 62]. Each specific DRG has an allocated CGW that relies on the average cost of inputs for medical procedures and diagnostic services required to achieve the appropriate patient outcome. The MalaysianDRG Casemix system will use base rate, CGW, hospital type, and other economic adjustors to derive the cost per case or the best-estimated cost for each DRG code for each respective year [62]. MOH MalaysianDRG Casemix costs by DRGs for inpatient care are the closest estimated costs since comprehensive cost information is not fully available [23].

For this study, a DRG code will be determined for each case using the MalaysianDRG Casemix application based on actual clinical diagnosis, procedures, and severity of illness. We will determine the casemix cost per case and casemix cost per patient day for every DRG code identified. FPP inpatient total operating costs will be calculated by adding casemix costs per DRG for all cases for the year.

#### Fixed and variable costs estimation of FPP inpatient service

Detailed information on hospital costs will be collected using specific codes according to Malaysian Treasury circular PS 1.1, Classification of Revenue and Expenditure Codes [59]. The circular guides and codes revenue and expenditure for all Federal Ministries and Departments. This circular ensures that the revenue and expenditure accounted for are accurate, uniform, and descriptive of the actual position in the Federal Government’s Financial Statements.

Financial codes are categorised into 14 cost items according to their financial descriptions in the circular. We defined variable costs as costs related directly to patient care occurring during the encounter and varying directly with the amount of resources provided. This study categorises five cost items as variable cost components: food and beverages, raw material supplies, medicines and medical supplies, other supplies, and purchased services. Instead, fixed costs remain the same regardless of patient numbers. The study classifies nine cost items as fixed costs, including staff emoluments, travel expenses, transportation of goods, communication and utilities, rental, maintenance, assets, grants and fixed payments. Based on these cost items, the proportion of fixed and variable costs for each hospital will be calculated from 2017 to 2020. Using the actual ratio of cost items classified as variable costs and fixed costs from each hospital’s annual total costs as a proxy, the cost per case for each DRG will be segregated into fixed and variable costs [63].

#### CVP analysis for the FPP inpatients service

The CVP analysis will be conducted after the FPP inpatient service volume, revenue, total costs, and fixed and variable costs have been determined. There are three commonly used approaches to illustrate the CVP analysis: the equation approach, the contribution margin approach, and the graphical approach [41]. The equation method and contribution margin method are useful to determine the profit at a specific level of volume of service delivered (e.g., the profit at 400 procedures performed). The graph method helps visualise the relationship between services delivered and profit or losses over a wide volume range [41].

This study will analyse CVP using the equation approach. The profit, contribution margin and break-even volume for the annual FPP inpatient service for each hospital and clinical department involved will be determined.

For the algebraic or equation method, the relationships that define the profit and costs in the CVP analysis can be stated as follows [30]:

P=TR−TC

TC=FC+VC

Where:

P = profit

TR = total revenues

TC = total costs

FC = fixed costs

VC = variable costs

The contribution margin and break-even point (BEP) in volume (number of patient days) can be calculated using the following formula [40]:

$$Break-even\;point\;in\;volume=\frac{FC}{UCM}$$

UCM=Revenueperunit−VCperunit

Where:

FC = fixed costs

UCM = unit contribution margin

VC = variable cost

The basic format of CVP analysis is meant for a single product. It means that the formulas above are all readily applicable to a single product firm or one with a non-varying sales mix. Most organisations, however, provide multiple services, such as a hospital offering various services. The CVP analysis for multiple products is computed for a hospital with multiple departments providing FPP inpatient services. In this case, the fixed costs must be divided by the weighted average unit contribution margin (WACM) to determine the BEP for the number of patient days [31], stated as follows:

$$Break-even\;point\;in\;volume=\frac{FC}{WACM}$$

Where:

FC = fixed costs

WACM = weighted average contribution margin

### Ethical consideration and dissemination

This study has been registered and ethically approved by the Medical Research and Ethics Committee of the Ministry of Health, Malaysia (NMRR-21-623-58791 (IIR)) and the Research Ethics Committee at the National University of Malaysia (Project code: FF-2021-295). This study will maintain the confidentiality of all data retrieved. Prior to the study, permission will be obtained from administrators and Clinical Research Centres of all selected FPP hospitals. A cover letter containing essential information and the purpose of the study will be sent to the directors of the selected hospitals, along with a permission form. A clarification will also be provided in the letter regarding the confidentiality and anonymity of the patient’s clinical and financial information. The results will be published in a relevant peer-reviewed scientific journal to ensure wide information dissemination. In addition, the study findings will be presented at professional meetings and communicated to hospitals, health managers, program owners, and policymakers within the Ministry of Health Malaysia.

### Data management

All clinical and financial data will be protected and anonymised by not identifying patients by name or other personal information. A numbering system will be employed to represent clinical records or any other documents containing patient information. The study data and documents will also be kept confidential, secured, and used only for research purposes. Study data and information relevant to service management for the FPP programme will be shared with stakeholders following the validation of the research. The study data will be stored in password-protected documents. Data access will be available to research personnel.

### Quality control measures

We will employ several methodological standards to ensure data quality with minimal bias, including quantitative study design and inclusion and exclusion criteria. The principal researcher will oversee data collection by directly communicating with the liaison officers from the selected FPP hospitals and the officers in charge of MalaysianDRG Casemix and MOH FPP Service at MOH headquarters. Based on the literature review and expert opinion, we have prepared the data collection forms in an Excel template (Microsoft® Excel® for Microsoft 365 MSO, Version 2205). Before constructing the Excel form, a data dictionary was developed that describes the variables in the data collection forms. This will streamline the data collection process and increase data accuracy and consistency. Data collectors and supervisors will receive detailed explanations and training. The collected data will be validated before the analysis to minimise the possibility of missing or incomplete data. Throughout the study, the principal researcher will review data integrity and quality.

### Data analysis

The data analysis will be conducted using statistical tools and a financial tool from management accounting, the cost volume profit (CVP) analysis. We will use the algebraic method to compute the profit and break-even point in volume. The CVP analysis results will be presented in table format. CVP analysis and break-even analysis will be performed using the Microsoft® Excel® for Microsoft 365 MSO, Version 2205 spreadsheet (Microsoft Corporation, USA). We will use descriptive statistics to summarise the data for age, gender, length of stay, diagnoses based on ICD-10 codes, procedures based on ICD-9-CM codes, DRG codes, volume of service based on per patient days and per discharge, revenues, and costs for inpatients. All independent and dependent variables will be subjected to normality tests. For all variables, the normality of data will be assessed using the Shapiro-Wilk test. Continuous variables will be reported as mean with standard deviation (if normally distributed) or median with interquartile range (if non-normality). Categorical variables will be reported as counts and percentages. The FPP inpatient volume, revenues, and costs trend will be visualised by plotting graphs to identify the data trend from 2017 to 2020. All statistical analysis will be performed using the International Business Machines Statistical Package for the Social Sciences (IBM® SPSS®) version 26.0 (IBM, USA). Statistical significance will be accepted at p-values < 0.05.

## Discussion

Even in developed countries such as Ireland, Australia, and the United Kingdom, DPH represents a significant source of extra revenue for public hospitals [5, 11, 12, 14]. Public hospitals rely on this revenue to sustain their services to public patients [11]. A portion of DPH revenue could be reinvested in public hospitals to improve healthcare quality and alleviate budgetary pressure [14]. National Health Service (NHS) in the UK, public hospitals have been encouraged to see private patients as a significant potential source of income to invest in NHS services [12, 14]. The Health and Social Care Act 2012 allows hospitals to earn more income from DPH from their overall NHS hospital trust income, which has increased from 2% to 49% since the act was passed [12].

However, studies have shown mixed results regarding DPH’s ability to generate additional revenues (or profits). Three health services in Australia derived more than $207 million (USD 135 million) from DPH between 2016 and 2018 [16]. However, neither the Department of Health and Human Services nor the audited health services know the cost of administering DPH. In addition, health services are unaware of how much billing, processing, and receipting private revenue costs them or how cost-effective it is to provide facilities, staff, and services to support private practice at no or low cost [16]. A different study found that all three district hospitals in Indonesia failed to recover their costs from the DPH [18]. Additionally, it has been reported in the UK that some, or all, of the DPH by NHS are operating at a loss or are generating bare minimum profits insufficient to justify their expenses [64].

Walpole et al. [14] highlighted the possibility of hidden cross subsidies because of the lack of proper accounting and recognition of the use of NHS facilities or equipment. These raise concerns regarding DPH’s financial consequences. Walpole [12] argued that the cost was not analysed adequately before the price was set, resulting in a loss for private patient care in a few public hospitals, while few other hospitals generated profits. Accounting skills are imperative in managing private patient care to prevent loss and thereby cause loss for public healthcare. Walpole [12] concluded that public hospitals should be required to measure, record, and report the costs and income from treating private patients to ensure adherence to public healthcare values, including equity of access and quality of care, and financial sustainability. The scarcity of healthcare resources should prompt healthcare managers and policymakers to pay closer attention to factors that may impact the sustainability of healthcare services, such as costs and revenues. To best serve the public interest, it is imperative for healthcare organisations, including public hospitals, to maintain the financial ability to meet their missions based on the economic insights of their programmes and services [32].

### Expected key results

This study provides insights into FPP services implementation in Malaysia through financial analysis. It will offer an overview of FPP services and the overall volume, revenue, and cost trend at four selected FPP hospitals from 2017 to 2020. The trend of these variables can be used to compare the performance of FPP services in each of the four selected hospitals. Furthermore, this study proposes a financial evaluation of the private care provision within public hospitals, focusing on break-even volume and profitability. The break-even volume for each hospital and clinical service will be among the results. The break-even volume and the volume of services delivered will allow us to assess if the hospitals or specific clinical services have delivered sufficient services so that total revenues equal total costs, which is the no-loss and no-profit threshold for FPP services. The break-even point (BEP) helps calculate the level of service that needs to be delivered before the organisation begins to generate profit. If the total number of patient days for services delivered exceeds the BEP, the hospital will gain profit, while a loss will occur if the service volume is below the BEP. Thus, this analysis will aid in decision-making on whether a particular volume of service will result in losses or profits. As the charges generate revenues, the CVP analysis can establish whether the FPP service has imposed appropriate charges. In addition, this study will provide information on the costs associated with FPP inpatient service delivery at the selected hospitals. FPP service administrators, policymakers, and regulators can better understand how volume, revenue, fixed costs, and variable costs influence operational profit.

### Strengths and limitations

To the best of our knowledge, this is the first study to quantify the profit or loss of MOH FPP inpatient services by evaluating the volume, costs (variable and fixed costs) and revenues impact towards profit vs loss on the MOH FPP service in Malaysia. This evaluation will demonstrate the application of CVP analysis in the healthcare field in the Malaysian context, explicitly utilising the MOH MalaysianDRG Casemix data. The secondary data will be collected from multiple sources, including four participating hospital-level and various national-level data sources, which are not freely accessible to the public. By utilising the datasets that had not previously been analysed, this study will be able to answer all the research questions and hence, the problem statement of this study. Furthermore, this study will illustrate the importance of cost and profit analysis in healthcare. Policymakers such as MOH can potentially utilise these results to develop future hospital standard costing procedures.

This research is subject to several limitations. First, this study is exploratory in nature. Thus, there is little guidance available from previous research regarding our approach of using cost volume profit (CVP) analysis to measure the impact of volume, revenue, and cost on the FPP inpatient service profit. We also anticipate challenges in identifying and verifying the variable and fixed costs for the inpatient FPP service cases. Despite the many advantages of the CVP analysis, this tool is limited by the restrictive assumptions underlying the CVP technique and can only estimate profit at best. Additionally, this study restricts the study setting to four hospitals across four regions in Malaysia based on the availability of complete MalaysianDRG Casemix system data. Thus, the outcomes may not be generalised to other public hospitals in Malaysia. However, these four hospitals have never been studied from the financial aspect before. This study will offer new info, especially the FPP financial impact from Cardiology and Cardiothoracic Surgery specialties in FPP services. In addition, the non-participation of public university hospitals with private patient wings and private hospitals may also limit the generalisability of the findings. Data accuracy will be another limitation of this study since it depends on the quality of data received at the hospital and national levels. DRG costing data are based on administrative records submitted by hospitals. However, the 2018 to 2020 audit report by Medical Development Division for four selected FPP hospitals has shown that all the hospitals have achieved 89% to 100% Casemix code classification accuracy. On top of that, MalaysianDRG Casemix data is the closest data to inpatient costing for treatment and procedures, which is readily available. Thus, the MOH MalaysianDRG Casemix data is found to be high in coding accuracy, and this is the most cost-efficient and time-saving methodology to gain cost information for all inpatient cases.

## Conclusion

CVP analysis can provide insightful and valuable information for program evaluation and decision-making. This is regardless of whether the program offers services for a minimal fee sufficient only to recover costs (i.e., to break-even) or whether the program services are being charged to generate a certain amount of revenue [65]. The application of this analysis to evaluate the financial performance of FPP services in MOH public hospitals may reveal valuable information on the effectiveness of FPP services in recouping service delivery costs and generating a surplus of revenue (profit). This study aims to give financial input and guidance to multiple stakeholders, including policymakers, healthcare professionals, and hospital administrators, in moulding a robust and strategic service expansion implementation. A comprehensive understanding of service volume, cost and pricing is imperative for making informed decisions to fulfil the objectives and ensure the sustainability of the FPP services.
